# Influence of Continuous Chelation on Root Dentin: A Laboratory Study

**DOI:** 10.3390/dj14100628

**Published:** 2026-10-02

**Authors:** Eva Correia, Inês Ferreira, Maria Ascensão Lopes, Ana Cristina Braga, Irene Pina-Vaz

**Affiliations:** 1 RISE-Health, Faculty of Dental Medicine, University of Porto, Rua Dr. Manuel Pereira da Silva, 4200-393 Porto, Portugaligvaz@fmd.up.pt (I.P.-V.); 2 REQUIMTE-LAQV, Department of Metallurgical and Materials Engineering, Faculty of Engineering, University of Porto, Rua Dr. Roberto Frias, 4200-465 Porto, Portugal; malopes@fe.up.pt; 3 ALGORITMI Center, Department of Production and Systems, University of Minho, Rua da Universidade, 4710-057 Braga, Portugal; acb@dps.uminho.pt

**Keywords:** continuous chelation, HEDP, sodium hypochlorite, EDTA, root dentin, dentin microhardness, surface tension, endodontic irrigation

## Abstract

**Background/Objectives:** Continuous chelation has emerged as an alternative to conventional sequential irrigation by combining sodium hypochlorite (NaOCl) with weak chelators in a single irrigant solution. This study evaluated and compared the effects of different final irrigation protocols on root dentin microhardness, chemical composition, and irrigant surface tension. **Methods:** Sixty extracted human canines were standardized for root length and canal lumen. Root dentin discs obtained from the middle third were randomly assigned to five groups according to the irrigation protocol: 3% NaOCl (total exposure time, 24 min), 17% EDTA (3 min), 3% NaOCl-DR HEDP (total exposure time, 24 min), 3% NaOCl/17% EDTA (NaOCl total exposure time, 24 min; EDTA 3 min), and 3% NaOCl/17% EDTA/3% NaOCl (NaOCl total exposure time, 24 min; EDTA 3 min). Dentin microhardness was assessed before and after treatment using the Vickers hardness test. Chemical composition was analyzed by energy-dispersive X-ray spectroscopy, and surface tension was measured using the pendant drop method. Data were analyzed using one-way ANOVA and Tamhane’s T2 post hoc test (α = 0.05). **Results:** Dentin microhardness decreased by approximately 4.0% with NaOCl-DR HEDP, 4.1% with EDTA alone, 14.3% with NaOCl, 16.9% with NaOCl/EDTA/NaOCl, and 39.7% with NaOCl/EDTA. Post hoc analysis showed that NaOCl/EDTA resulted in significantly greater reductions in dentin microhardness than EDTA alone, continuous chelation with NaOCl-DR HEDP, and the NaOCl/EDTA/NaOCl protocol (*p* < 0.05). EDTA and NaOCl-DR HEDP exhibited the smallest reductions in microhardness and did not differ significantly from each other. Regarding chemical composition, NaOCl-DR HEDP induced intermediate changes in organic and inorganic components. NaOCl alone yielded the lowest carbon content, while EDTA and NaOCl/EDTA sequence showed the lowest calcium and phosphorus levels. Surface tension was highest for the H_2_O-HEDP solution, followed by 3% NaOCl-DR HEDP and 17% EDTA, and lowest for 3% NaOCl. **Conclusions:** Despite its higher surface tension, the continuous chelation protocol with NaOCl-DR HEDP preserved dentin microhardness to a similar extent as EDTA alone and promoted more moderate changes in dentin chemical composition than the conventional final irrigation protocols, particularly NaOCl/EDTA, suggesting a conservative effect on root dentin.

## 1. Introduction

The increased susceptibility of endodontically treated teeth to fracture is multifactorial, involving intrinsic dentin characteristics, restorative procedures, and chemical factors such as intracanal medicaments and irrigants [1]. Root canal debridement and disinfection rely on chemomechanical preparation, combining instrumentation with chemical irrigation. An ideal irrigant should provide broad antimicrobial activity, dissolve organic tissue, neutralize endotoxins, and manage smear layer formation while preserving dentin structure [2]. Sodium hypochlorite (NaOCl) remains the gold standard irrigant due to its broad antimicrobial activity and tissue-dissolving capacity, fulfilling most of the requirements of an ideal irrigant [3]. Its main limitation is the absence of a decalcifying effect, preventing effective removal of hard-tissue debris and the inorganic fraction of the smear layer, which may reduce dentinal tubule penetration and disinfection efficacy [4,5]. Moreover, NaOCl has been associated with alterations in dentin properties, including reductions in elasticity, strength, and microhardness [2,6,7]. To overcome its inability to remove the inorganic smear layer, chelating agents such as ethylenediaminetetraacetic acid (EDTA) are commonly used as adjuncts to NaOCl [2]. Because EDTA interacts chemically with NaOCl, the two solutions must be applied sequentially rather than simultaneously [2,5]. Although this protocol improves smear layer removal, it is time-consuming and has been associated with dentin demineralization and erosion, as demonstrated by SEM studies, potentially compromising the mechanical properties of dentin [2,8].

Following smear layer removal with EDTA, a final rinse with NaOCl has been advocated (NaOCl/EDTA/NaOCl) to eliminate residual chelator, improve penetration into dentinal tubules, and enhance biofilm disruption [2]. Nevertheless, concerns remain regarding collagen degradation and erosion of the root canal walls caused by renewed NaOCl exposure [9,10], although the clinical significance of these changes has yet to be established [11].

Continuous chelation has emerged as an alternative to conventional sequential irrigation by combining NaOCl with weak chelators such as etidronate (a salt of 1-hydroxyethylidene-1,1-diphosphonic acid; HEDP) in a single solution. HEDP can be mixed with NaOCl with minimal short-term reduction in free available chlorine [12,13] and promotes gradual hard-tissue and smear layer removal, although less effectively than EDTA [14].

Commercial formulations in which HEDP is used as an additive to NaOCl, such as Dual Rinse HEDP (Medcem, Vienna, Austria; DR HEDP), are clinically available and have been reported to preserve the desirable properties of NaOCl while simplifying irrigation protocols and improving shaping efficiency [15,16]. Nevertheless, differences in study methodologies and outcome measures continue to limit direct comparisons and the overall interpretation of the available evidence [16].

Dentin is a composite tissue composed of approximately 22% organic material by weight, predominantly type I collagen, embedded within a hydroxyapatite-based inorganic matrix [17]. This hierarchical arrangement, together with its intrinsic water content, is responsible for dentin’s mechanical properties, including strength, toughness, and resistance to deformation [1]. During endodontic treatment, dentin is conditioned by endodontic irrigants, including proteolytic and demineralizing agents, which may affect both the collagen network and the mineralized matrix [17]. While essential for root canal disinfection and cleaning, these irrigants may modify dentin structure and adversely affect properties such as microhardness, with potential implications for its mechanical performance [11,18,19].

Dentin microhardness reflects resistance to localized deformation and is commonly used as an indirect indicator of mineral content and structural integrity [20]. Although differences in experimental conditions, particularly the indentation method employed (Knoop, Vickers, or nanoindentation) and NaOCl exposure time, may partly account for discrepancies among studies, the effects of continuous chelation on dentin microhardness remain inconclusive [15,18].

Elemental analysis, commonly performed using energy-dispersive spectroscopy (EDS), provides additional insight into the effects of irrigation protocols on dentin structure and composition, which may ultimately influence its mechanical properties [7,21]. Nevertheless, the limited number of available studies, together with variations in study designs and exposure protocols, hinders direct comparisons and limits the clinical extrapolation of the available evidence.

Surface tension is another relevant physicochemical property of endodontic irrigants, as lower values enhance wettability, facilitating irrigant penetration and potentially improving root canal disinfection [22,23]. Increased wettability, being associated with deeper penetration, has the potential to reduce dentin microhardness [11,15]. Interestingly, the addition of HEDP to NaOCl has been reported to increase surface tension [24,25], which contrasts with the commonly accepted preference for low-surface-tension irrigants to enhance wettability and penetration. However, the implications of this effect for dentin properties remain uncertain.

Therefore, the primary aim of this laboratory study was to evaluate the effect of continuous chelation with NaOCl-DR HEDP solution on root canal dentin microhardness, compared with conventional sequential irrigation protocols, namely NaOCl/EDTA and NaOCl/EDTA/NaOCl. A secondary objective was to assess changes in the elemental composition of radicular dentin following exposure to these irrigation regimens. In addition, the surface tension of NaOCl and HEDP, used alone and in combination (NaOCl-DR HEDP), was evaluated to further characterize the physicochemical behaviour of the continuous chelation system.

The null hypotheses tested were: (1) continuous chelation with NaOCl-DR HEDP and sequential irrigation protocols (NaOCl/EDTA and NaOCl/EDTA/NaOCl) result in similar reductions in dentin microhardness; (2) no differences exist in the elemental composition of radicular dentin following exposure to the different irrigation protocols; and (3) NaOCl and DR HEDP exhibit similar surface tension values when used individually or in combination.

## 2. Materials and Methods

All experiments in this study using human extracted teeth were approved by the Ethics Committee of the Faculty of Dental Medicine, University of Porto (approval no. 28/2024). 

### 2.1. Specimens Selection and Preparation

Teeth were obtained from patients attending the clinics of the Faculty of Dental Medicine, University of Porto, and were stored in distilled water at 5 °C until use. Donor age was not available because all specimens were anonymized prior to inclusion in the study.

Ninety-six extracted human canines with a single root canal, radiographically confirmed, were selected. Teeth with previous endodontic treatment, root caries, dilacerations, anatomical morphological deformities, resorptions, immature apices, cracks, calcifications, and fractures were excluded. The teeth were decoronated, and the roots standardized to 16 mm. All visible soft and hard tissues adherent to the roots were removed with curettes, and the specimens were disinfected in 1% NaOCl for 24 h. Working length was determined by inserting a size 10 K-file into the canal until its tip was visible at the apical foramen and subtracting 1 mm from this length. Root canals were prepared using the ProTaper Gold system (Dentsply Sirona, Ballaigues, Switzerland) up to size F3 at working length, with distilled water as the irrigant. Each canal was irrigated with 5 mL of distilled water before instrumentation and between each file. Standardization of the canal lumen was performed with Gates-Glidden drills (sizes 1–4; Dentsply Sirona), encompassing the entire middle third. After drying the canals with paper points, the roots were sectioned under refrigeration to obtain 2 mm thick discs from the middle third of the prepared teeth (untreated control) and polished. Sixty dentin discs were allocated to the microhardness experiment and randomly assigned to five experimental groups (n = 12), as described in the following section. For EDS analysis, 36 additional dentin discs were prepared using the same protocol and allocated to six groups (n = 6), comprising the five experimental groups and an untreated control group; these specimens constituted a dedicated subsample that was distinct from those used for microhardness testing.

### 2.2. Irrigation Protocols

The following irrigating solutions were tested:

- Sodium hypochlorite 3% (Coltene, Whaledent AG, Altstatten, Switzerland).
- 17% EDTA (Coltene, Whaledent AG, Altstatten, Switzerland).
- Dual Rinse^®^ HEDP (Dual Rinse HEDP, Medcem, Vienna, Austria).

All solutions were stored at 5 °C between experiments; prior to being used, they were removed from the refrigerator and stored for 60 min at room temperature. The mixed 3% NaOCl-DR HEDP solution was obtained by adding 10 mL of 3% NaOCl to 0.9 g of DR HEDP powder (mean content per capsule) as recommended by the manufacturer (https://www.medcem.ch/en/products/dual-rinsehedp/use.html; (accessed on 14 June 2026)).

Specimens were then randomly numbered and divided into five experimental groups (n = 12) and immersed in the respective solutions according to the following irrigation protocols, with exposure times simulating clinical conditions [26]:

- Group 1: 3% NaOCl (6 mL/21 min) + distilled water (3 mL/3 min) + 3% NaOCl (3 mL/3 min) + distilled water (3 mL/3 min).
- Group 2: distilled water (6 mL/21 min) + EDTA (3 mL/3 min) + distilled water (3 mL/3 min) + distilled water (3 mL/3 min).
- Group 3: NaOCl-DR HEDP (6 mL/21 min) + distilled water (3 mL/3 min) + NaOCl-DR HEDP (3 mL/3 min) + distilled water (3 mL/3 min).
- Group 4: 3% NaOCl (6 mL/21 min) → distilled water (3 mL/3 min) → 3% NaOCl (3 mL/3 min) → 17% EDTA (3 mL/3 min) → distilled water (3 mL/3 min).
- Group 5: 3% NaOCl (6 mL/21 min) → 17% EDTA (3 mL/3 min) → 3% NaOCl (3 mL/3 min) → distilled water (3 mL/3 min).

After the irrigation protocols had been fully defined, abbreviated group labels were used for clarity and consistency, while preserving the corresponding irrigation sequence, as follows:

G1: NaOCl (NaOCl → H_2_O → NaOCl → H_2_O).
G2: EDTA (H_2_O → EDTA → H_2_O → H_2_O).
G3: NaOCl-DR HEDP (NaOCl-DR HEDP → H_2_O → NaOCl-DR HEDP → H_2_O).
G4: NaOCl/EDTA (NaOCl → H_2_O → NaOCl → EDTA → H_2_O).
G5: NaOCl/EDTA/NaOCl (NaOCl → EDTA → NaOCl → H_2_O).

**Figure 1:**
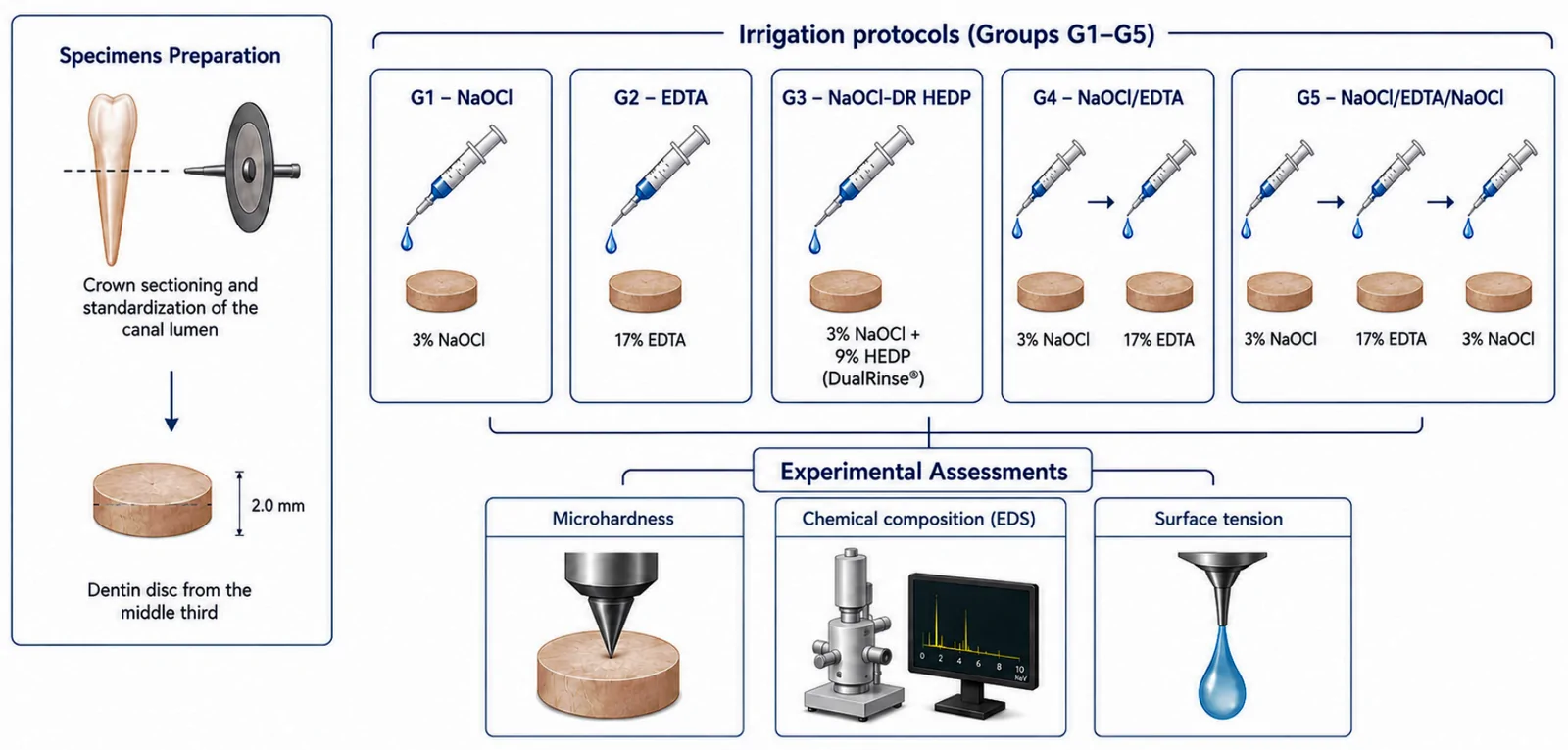
Schematic representation of specimen preparation, irrigation protocols and experimental assessments.

A schematic overview of specimen preparation, irrigation protocols, and experimental assessments is presented in Figure 1.

### 2.3. Microhardness Measurement

Microhardness was assessed both before and after immersion in the irrigation protocols, using a Falcon 400G2 Vickers Hardness Tester (Innovatest, Maastricht, The Netherlands), and values were expressed as Vickers Hardness Number (HV). Three indentations were made per slice, at 100 μm from the canal lumen, using a 50 g load for 15 s. Following irrigation protocols, three additional indentations were performed on the opposite, symmetrical side of each specimen. The baseline indentations remained visible throughout the experiment and were used as a reference for positioning the post-treatment indentations. Post-treatment measurements were performed at the same standardized distance (100 μm) from the canal lumen in the corresponding symmetrical region of each specimen. Variations between initial and final microhardness values were calculated, using the baseline measurements of each specimen as the control. Sample size calculation was performed a priori using G*Power software (version 3.1.9.7, Heinrich Heine University, Düsseldorf, Germany). Based on a repeated-measures ANOVA (within-factors design), assuming an effect size of f = 0.25, a significance level of 5% (α = 0.05), a statistical power of 95% (1 − β = 0.95), five groups, and two measurements, the minimum required sample size was 55 specimens. To compensate for potential specimen loss, 60 samples were included in the study.

### 2.4. Elemental Composition Analysis

Energy dispersive X-ray spectroscopy (EDS; Model FEI Quanta 400 FEG ESEM/EDAX Genesis X4M; FEI Company, Hillsboro, OR, USA) was used to perform a semiquantitative analysis of compositional changes in the middle-third root dentin. The atomic percentages of the different elements were measured in triplicate. Based on the methodology described by Rath et al. [21] six dentin discs per group were selected for EDS analysis, coated with a Au/Pd thin film by sputtering using an SPI Module Sputter Coater. The relative atomic percentages of carbon (C), oxygen (O), phosphorus (P), calcium (Ca), and magnesium (Mg) were determined, and changes in the Ca/P ratio were also assessed. A non-treated control group was included exclusively for EDS analysis; these specimens were immersed in distilled water for 30 min and used as a baseline for compositional comparisons.

### 2.5. Surface Tension Evaluation

Surface tension was measured for four irrigants: 3% NaOCl, 17% EDTA, DR HEDP dissolved in distilled water (H_2_O-DR HEDP), and 3% NaOCl-DR HEDP. All solutions were kept at room temperature prior to testing, and all measurements were performed at approximately 23 °C. A video-based optical contact angle measurement system (OCA 15 Plus, DataPhysics Instruments GmbH, Filderstadt, Germany) equipped with an electronic dosing unit was used. Surface tension was determined by pendant drop shape analysis using the Young-Laplace model implemented in the SCA20 software (DataPhysics Instruments GmbH, Germany; version 2.0). For each irrigant, four independent droplets of 4 μL were dispensed, video-imaged, and analyzed. All measurements were performed by a blinded operator under standardized environmental conditions.

### 2.6. Statistical Analysis

Statistical analysis was performed using SPSS software (IBM SPSS Statistics 30.0.0). Homogeneity and normality of the data were assessed using Levene’s test and the Kolmogorov–Smirnov test, respectively. One-way ANOVA followed by Tamhane’s T2 post hoc test was used for the analysis of microhardness, surface tension, and chemical composition data. A significance level of 5% was adopted (*p* < 0.05). For the microhardness analysis, a variable (“dif”) was created to represent the difference between the initial and final microhardness values, thereby quantifying the change in microhardness.

## 3. Results

### 3.1. Microhardness Assessment

**Figure 2:**
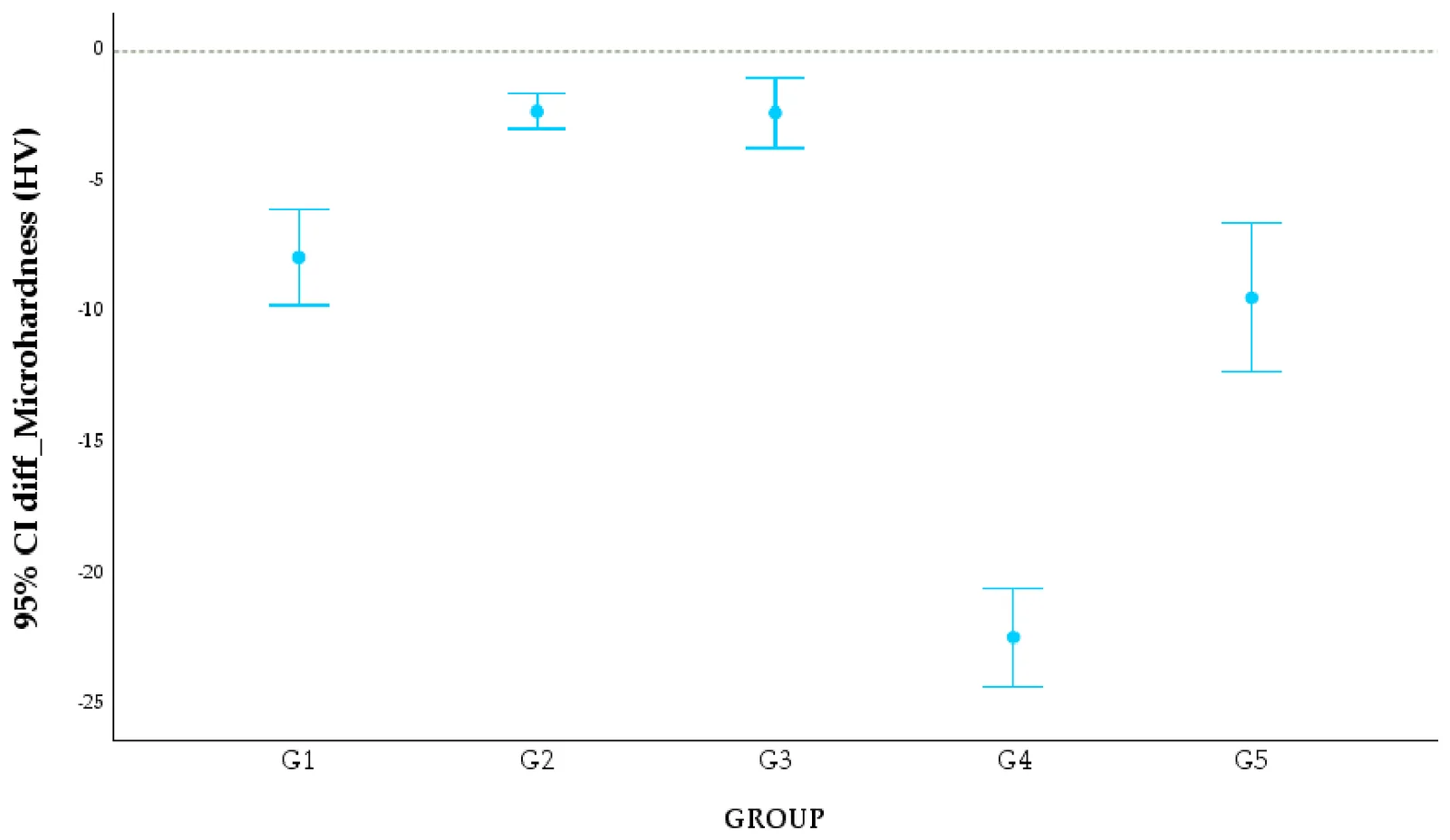
Mean change in dentin Vickers microhardness (HV) after exposure to the different irrigation protocols. Data are presented as mean values and 95% confidence intervals (CIs).

ANOVA revealed statistically significant differences in mean microhardness variation among the groups (F(4,55) = 93.997, *p* < 0.001, η^2^ = 0.872). Dentin microhardness decreased by approximately 4.0% with NaOCl-DR HEDP, 4.1% with EDTA alone, 14.3% with NaOCl, 16.9% with NaOCl/EDTA/NaOCl, and 39.7% with NaOCl/EDTA (Figure 2). Post hoc comparisons showed that all groups differed significantly from each other (*p* < 0.001), except for Group 1 (NaOCl) and Group 5 (NaOCl/EDTA/NaOCl), which did not present a statistically significant difference (*p* = 0.982), and Group 2 (EDTA) and Group 3 (NaOCl-DR HEDP) (*p* = 1.000).

Table 1 presents the baseline and post-treatment dentin microhardness values, as well as the mean microhardness change observed after each irrigation protocol.

### 3.2. Elemental Composition Analysis

The semiquantitative EDS analysis revealed significant differences in the relative atomic percentages of dentin elements among the irrigation protocols (*p* < 0.05 for all elements analyzed), Table 2.

Carbon content differed significantly among groups (F(5,30) = 91.83, *p* < 0.001, η^2^ = 0.939). The highest values were observed in G2 (EDTA), whereas the lowest values were found in G1 (NaOCl). Intermediate values were observed in G3 (NaOCl-DR HEDP), G5 (NaOCl/EDTA/NaOCl), and the control group, indicating a more balanced organic composition.

Oxygen levels also varied significantly among the experimental groups (F(5,30) = 98.17, *p* < 0.001, η^2^ = 0.942). G2 (EDTA) exhibited the lowest oxygen content, while higher values were observed in G1 (NaOCl), G3 (NaOCl-DR HEDP), and the control group, suggesting greater preservation of the mineral phase in these groups. G4 (NaOCl/EDTA) and G5 (NaOCl/EDTA/NaOCl) showed intermediate oxygen levels.

Significant differences were observed for both phosphorus (F(5,30) = 37.11, *p* < 0.001, η^2^ = 0.861) and calcium (F(5,30) = 40.17, *p* < 0.001, η^2^ = 0.870). The lowest phosphorus and calcium contents were recorded in G2 (EDTA), whereas significantly higher values were observed in G1 (NaOCl) and G5 (NaOCl/EDTA/NaOCl), with G3 (NaOCl-DR HEDP) showing intermediate values. G4 (NaOCl/EDTA) demonstrated a significant reduction in both calcium and phosphorus compared with G1 (NaOCl) and G5 (NaOCl/EDTA/NaOCl).

Magnesium content showed less pronounced variations among groups, although overall differences reached statistical significance (F(5,30) = 5.13, *p* = 0.002, η^2^ = 0.461). G5 (NaOCl/EDTA/NaOCl) exhibited significantly higher magnesium levels than G1 (NaOCl) and G2 (EDTA), while no significant differences were observed among the remaining groups.

The Ca/P ratio also differed significantly among the irrigation protocols (F(5,30) = 15.68, *p* < 0.001, η^2^ = 0.723). The lowest ratio was observed in G3 (NaOCl-DR HEDP), whereas G4 (NaOCl/EDTA) exhibited the highest values. G1 (NaOCl), G5 (NaOCl/EDTA/NaOCl), and the control group demonstrated comparable Ca/P ratios, with no statistically significant differences between them.

Post hoc comparisons revealed that G2 (EDTA) differed significantly from most other groups for carbon, phosphorus, and calcium, indicating a distinct compositional profile characterized by increased organic content and reduced mineral content. In contrast, G1(NaOCl), G5 (NaOCl/EDTA/NaOCl), and the control group formed a homogeneous subset for several elements, suggesting similar preservation of dentin mineral composition. The continuous chelation protocol, G3 (NaOCl-DR HEDP), showed a compositional profile comparable to mineral-preserving groups for several parameters, while maintaining significantly lower Ca/P values.

### 3.3. Surface Tension Evaluation

There were statistically significant differences in relation to all solutions regarding the measurement of surface tension (ANOVA, F(3,12) = 113.236, *p* < 0.001, η^2^ = 0.966). The surface tension of 3% NaOCl was lower compared to DR HEDP dissolved in distilled water (H_2_O-DR HEDP) and 3% NaOCl-DR HEDP (Table 3).

Post hoc comparisons showed that all pairwise differences were statistically significant (F(3,12) = 113.24, *p* < 0.001). Surface tension values were highest for the H_2_O-DR HEDP solution, followed by 3% NaOCl-DR HEDP, then 17% EDTA, and were lowest for 3% NaOCl. In practical terms, adding DR HEDP produced the greatest increase in surface tension when dissolved in water and also raised the surface tension when combined with 3% NaOCl, whereas NaOCl alone exhibited the lowest surface tension among the tested irrigants.

## 4. Discussion

Continuous chelation, achieved by simultaneously using a weak chelator combined with NaOCl throughout root canal preparation, has been proposed as an alternative to the conventional sequential irrigation with NaOCl and EDTA, aiming to overcome its limitations without compromising key chemical properties. The present study evaluated the effect of continuous chelation on root canal dentin microhardness, using a commercially available system, DR HEDP, in which 0.9 g of etidronate was mixed with 10 mL of 3% NaOCl solution immediately before use.

The findings demonstrated that commonly used irrigating solutions affect dentin mechanical properties—particularly microhardness—depending on their chemical composition and application sequence. Continuous chelation (G3) and EDTA alone (G2) resulted in the smallest reductions in dentin microhardness, with no significant differences between them. In contrast, the conventional sequential protocol (NaOCl/EDTA) produced the greatest decrease in microhardness, exceeding that observed with individual irrigants (NaOCl or EDTA), continuous chelation, and the NaOCl/EDTA/NaOCl sequence.

This pattern also aligns with evidence showing that reductions in dentin microhardness are related to the chelating strength of the irrigant, with weaker agents inducing less structural alteration than stronger ones [18]. Accordingly, HEDP, a mild chelator used as an irrigation additive to NaOCl, resulted in less dentin softening than the conventional sequential NaOCl/EDTA protocol (G4). This trend has been consistently reported in studies demonstrating greater dentin degradation and reduced microhardness with more aggressive chelating regimens [14,15,27,28].

From a mechanistic perspective, dentinal collagen is normally protected by a mineral phase. NaOCl, due to its low molecular weight, can penetrate mineralized collagen and promote partial degradation, although hydroxyapatite crystals act as a barrier limiting its effect [18,29]. Subsequent EDTA application removes this inorganic layer, exposing collagen fibrils and leading to more pronounced structural damage [21]. Ultrastructural analyses suggest that sequential chelation promotes deeper subsurface disruption, whereas continuous chelation preserves partially mineralized collagen, maintaining a more balanced organic–inorganic structure and higher microhardness [21].

Corroborating a previous study [21], the semiquantitative EDS analysis showed that dentin treated with NaOCl-DR HEDP (G3) exhibited lower carbon content than the NaOCl/EDTA sequence (G4), consistent with the sustained deproteinizing effect of NaOCl during continuous chelation. Oxygen atomic percentages were also maintained in G3, as in the NaOCl-only group (G1), suggesting preservation of the mineral phase. In contrast, G2 (EDTA) and G4 (NaOCl/EDTA) showed lower calcium and phosphorus atomic percentages, whereas G3 retained significantly higher levels, indicating greater mineral preservation with etidronate, likely due to its weaker chelating action. Although continuous chelation resulted in slightly lower Ca/P ratios, overall variations were small, suggesting proportional changes in calcium and phosphorus. This finding is consistent with Marques et al. [7], who reported that chelating protocols may affect the Ca/P balance without substantial mineral loss. Therefore, the lower Ca/P ratio observed with NaOCl-DR HEDP may reflect relative changes in calcium and phosphorus distribution and a subtle shift in ionic balance. Magnesium, a minor constituent of dentin apatite, remained relatively stable across groups, indicating that the mineral changes induced by the irrigation protocols primarily involved calcium and phosphorus rather than a generalized depletion of the mineral phase.

In the present study, a final NaOCl flush after EDTA (G5) partially restored dentin microhardness to levels comparable to those obtained with NaOCl alone, suggesting a partial re-establishment of the organic–inorganic balance. Supporting this interpretation, C values in G5 were not significantly different from those in the NaOCl-only group (G1) and were significantly lower than those observed in G4 (NaOCl/EDTA). Accordingly, the relative phosphorus and calcium atomic percentages in G5 were significantly higher than those observed with the conventional NaOCl/EDTA protocol (G4). Consistent with these findings, a study using chlorhexidine (CHX) as a final irrigant instead of NaOCl has reported dentin chemical profiles comparable to those observed in the NaOCl/EDTA/NaOCl sequence, suggesting that the nature of the final irrigant may play a key role in modulating the surface chemical composition of dentin [7].

This finding contrasts with previous reports [28], which, despite confirming differences in microhardness among irrigating solutions, found no significant effect of a final NaOCl rinse under a similar experimental design. These discrepancies may be explained by methodological differences, particularly in the indentation techniques used: while Tartari et al. [28] employed the Knoop method, the present study used Vickers microhardness testing, which involves greater penetration depth, typically reaching approximately two to three times deeper than Knoop indentations under comparable testing conditions, and may therefore be more sensitive to subsurface changes induced by NaOCl [6].

Overall, the present findings suggest that the relative elemental profile of dentin is influenced by both the chelating strength of the irrigant and the irrigation sequence. EDTA-based protocols were associated with lower relative calcium and phosphorus atomic percentages, whereas NaOCl and NaOCl-DR HEDP protocols exhibited elemental profiles suggestive of greater preservation of the mineral phase. Importantly, these observations should not be extrapolated to all combined irrigant formulations. Different all-in-one systems may produce distinct demineralizing effects on dentin depending on their composition [30]. Therefore, the effects observed with one formulation should not be assumed to be representative of all all-in-one systems.

This study also assessed the surface tension of NaOCl and DR-HEDP, individually and in combination. The NaOCl-DR HEDP mixture exhibited higher surface tension than NaOCl or EDTA alone, corroborating previous findings [24]. As higher surface tension is generally associated with reduced wettability and penetration ability, the addition of DR HEDP would be expected to decrease solution spreading on dentin surfaces. Nevertheless, despite its higher surface tension, the NaOCl-DR HEDP protocol was associated with greater preservation of dentin microhardness and relative elemental composition than the EDTA-based protocols. Therefore, the observed effects on dentin are more likely related to the milder chelating action of HEDP and its chemical compatibility with NaOCl than to differences in surface tension.

Overall, the NaOCl-DR HEDP system appears to be a promising alternative to conventional irrigation protocols. By combining antimicrobial, proteolytic, and mild chelating actions in a single solution, it enables continuous chelation throughout instrumentation, reducing smear layer and debris formation while simplifying the irrigation protocol. In addition, the chemical compatibility between etidronate and NaOCl limits excessive demineralization, which may explain the smaller reduction in microhardness observed in the present study [16,30,31]. The benefits of continuous chelation may also extend beyond microhardness, as improvements in other mechanical properties, including flexural strength and fracture resistance, have been reported [18].

This study has several limitations that should be considered when interpreting the findings. As an in vitro investigation, it does not fully reproduce clinical conditions, which may limit the direct applicability of the results. Moreover, the analysis was restricted to middle-third dentin from extracted human single-rooted canines; therefore, the findings may not be directly generalizable to apical dentin or teeth with more complex root canal anatomies. In addition, owing to the heterogeneous nature of dentin, microhardness measurements mainly reflect surface changes and provide only limited information about the bulk material. Furthermore, post-treatment indentations were performed on the opposite symmetrical side of each disc rather than at the exact baseline location, which may have introduced some variability; however, this approach has been adopted in a previous study [6]. Other factors that may influence dentin mechanical behavior, such as individual dentin characteristics, instrumentation protocols, remaining dentin thickness, and donor-related variables (e.g., age), were not assessed.

Additionally, the sample size calculation was based on the primary outcome (microhardness) and was not specifically designed to power the secondary EDS and surface tension analyses, an approach frequently adopted in previous in vitro studies evaluating multiple complementary outcomes [21,32]. Finally, although microhardness was selected as the primary outcome in accordance with previous studies [15,28,33], changes in microhardness should be interpreted cautiously and in conjunction with other structural and mechanical parameters.

Despite these limitations, efforts were made to enhance the clinical relevance of the experimental design. Irrigants were applied using exposure times consistent with those encountered in endodontic practice, and the middle third of the root was selected for analysis to reduce variability in dentinal tubule density, in accordance with established methodologies [18,21].

## 5. Conclusions

Within the limitations of this study, the NaOCl-DR HEDP protocol was associated with less pronounced reductions in dentin microhardness than the NaOCl/EDTA protocol and preserved dentin microhardness to a similar extent as EDTA alone. Furthermore, it promoted more moderate changes in dentin elemental composition than the conventional sequential irrigation protocols. These findings suggest that NaOCl-DR HEDP may represent a more conservative irrigation approach with respect to selected physicochemical properties of root dentin.

The semiquantitative EDS analysis suggested a more mineralized chemical profile following continuous chelation, characterized by higher relative calcium and phosphorus atomic percentages and less evidence of mineral alteration than that observed after EDTA-based irrigation groups (EDTA alone and NaOCl/EDTA). Overall, these findings highlight the significant influence of irrigation strategies on the chemical composition and ultrastructural organization of radicular dentin. In particular, sequential irrigation with NaOCl/EDTA appears to promote more pronounced mineral loss, whereas continuous chelation with NaOCl-DR HEDP better maintains dentin mineral balance, suggesting that the milder chelating action of HEDP may outweigh the effect of its longer exposure time. Further well-designed, multidisciplinary studies under clinically relevant conditions are required to confirm these findings and establish their clinical relevance.

## Figures and Tables

**Table 1:** Baseline and post-treatment dentin microhardness values (HV) according to the irrigation protocol. Data are presented as mean ± standard deviation (SD).

Group	Baseline HV (Mean ± SD)	Post-Treatment HV (Mean ± SD)	Change in HV (Mean ± SD)
G1	55.34 ± 1.12	47.44 ± 2.70	−7.90 ± 2.90
G2	55.92 ± 0.29	53.61 ± 1.24	−2.31 ± 1.07
G3	56.57 ± 1.50	54.21 ± 1.76	−2.36 ± 2.11
G4	56.49 ± 0.90	34.05 ± 2.85	−22.44 ± 2.98
G5	56.03 ± 0.65	46.58 ± 4.63	−9.44 ± 4.51

**Table 2:** Mean ± standard deviation of relative atomic percentages (%) from elemental analysis (* The control group consisted of samples immersed in distilled water for 30 min).

Group	Carbon (C)	Oxygen (O)	Magnesium (Mg)	Phosphorus (P)	Calcium (Ca)	Ca/P Ratio
G1	11.38 ± 0.45	46.94 ± 1.83	0.59 ± 0.06	14.78 ± 0.46	25.14 ± 1.36	1.70 ± 0.05
G2	43.47 ± 4.44	33.73 ± 1.94	0.52 ± 0.12	7.94 ± 1.51	12.91 ± 2.33	1.63 ± 0.06
G3	15.28 ± 1.20	48.13 ± 1.00	0.72 ± 0.15	13.82 ± 0.53	21.00 ± 0.85	1.52 ± 0.02
G4	32.08 ± 5.53	39.10 ± 0.80	0.59 ± 0.15	9.15 ± 2.28	15.57 ± 3.41	1.71 ± 0.06
G5	16.90 ± 2.74	43.81 ± 1.04	0.81 ± 0.08	13.93 ± 0.65	23.42 ± 1.35	1.68 ± 0.02
Control *	15.43 ± 1.55	46.10 ± 1.18	0.52 ± 0.16	14.20 ± 0.27	23.43 ± 0.35	1.65 ± 0.02

**Table 3:** Surface tension (mJ/m^2^) of the irrigating solutions (SD—standard deviation).

Irrigating Solution	Surface Tension Mean Value ± SD
3% NaOCl	51.02 ± 2.25
17% EDTA	60.75 ± 0.21
H_2_O-DR HEDP	65.21 ± 0.47
3% NaOCl-DR HEDP	62.29 ± 0.12

## Data Availability

The data presented in this study are available from the corresponding author upon reasonable request.
